# Tattoo ink nanoparticles in skin tissue and fibroblasts

**DOI:** 10.3762/bjnano.6.120

**Published:** 2015-05-20

**Authors:** Colin A Grant, Peter C Twigg, Richard Baker, Desmond J Tobin

**Affiliations:** 1 Advanced Materials Engineering, Faculty of Engineering and Informatics, University of Bradford, Bradford, BD7 1DP, United Kingdom; 2 Centre for Skin Sciences, Faculty of Life Sciences, University of Bradford, Bradford, BD7 1DP, United Kingdom

**Keywords:** atomic force microscopy (AFM), dermis, nanoparticles, skin, tattoo ink

## Abstract

Tattooing has long been practised in various societies all around the world and is becoming increasingly common and widespread in the West. Tattoo ink suspensions unquestionably contain pigments composed of nanoparticles, i.e., particles of sub-100 nm dimensions. It is widely acknowledged that nanoparticles have higher levels of chemical activity than their larger particle equivalents. However, assessment of the toxicity of tattoo inks has been the subject of little research and ink manufacturers are not obliged to disclose the exact composition of their products. This study examines tattoo ink particles in two fundamental skin components at the nanometre level. We use atomic force microscopy and light microscopy to examine cryosections of tattooed skin, exploring the collagen fibril networks in the dermis that contain ink nanoparticles. Further, we culture fibroblasts in diluted tattoo ink to explore both the immediate impact of ink pigment on cell viability and also to observe the interaction between particles and the cells.

## Introduction

The act of tattooing has been practised for many centuries in a number of countries including Japan, China, New Zealand as well as in regions of North Africa. The oldest recorded human tattoo was found on a well-preserved natural mummy from about 5,300 years ago, found in the Ötztal Alps in Italy, close to the border with Austria [1]. These ancient tattoos do not appear to have had decorative importance but may had have had some medical/therapeutic relevance, some appear to be close to traditional acupuncture points [2]. Today, tattooing is becoming increasingly popular across several sections of society, with increasing numbers of tattoo parlours opening for business. However, despite this striking cultural shift we know very little about the biochemical reactivity of ink particles with skin cells and tissues (including some of the key constituent components, e.g., fibroblasts and associated collagen fibrillar networks).

The tattooing process involves inserting ink pigment of the desired colour into the dermis layer of the skin. This is carried out by first dipping a needled tattoo instrument into the coloured ink before applying to the skin. The oscillating ink-coated needle punctures the skin in the range of 100 times per second, depositing the ink pigments 1.5 to 2 mm below the skin surface. Thus, the needle penetrates the skin through the epidermis and into the papillary layer of the dermis, where the ink particles accumulate. As with any type of trauma to the dermis, the first response of the body is to stop the resultant bleeding to form a clot. Then the skin tissue swells (edema) followed by a migration of immune system cells to the wound site (neutrophils and macrophages) in order to phagocytose foreign substances, cell debris and microbes. Any damaged collagen in the wounded papillary dermis is then repaired through the action of fibroblasts, ultimately laying down scar tissue. Over long periods of time the tattoo ink particles can be found to gradually move to the deeper dermis (i.e., reticular dermis), which gives the tattoo a faded and blurred appearance. Importantly, after tattoo ink insertion associated pigment particles can be found to leave the skin via its vasculature and enter the lymphatic system (nodes) [3].

Tattoo inks are commonly made up of a mixture of small organic pigments, water and isopropyl alcohol. Surprisingly, manufacturers of tattoo ink are not compelled to reveal the precise ingredients or chemical composition of their ink products despite their potential systemic absorption. Black inks are commonly made from soot (carbon black) particles. Tattoo inks can contain polycyclic aromatic hydrocarbons (PAHs) at a range of concentrations, which are reported to be carcinogenic, mutagenic and could pose other health risks to the skin [4]. Further, it was recently reported that tattooed young individuals can exhibit adverse reactions, especially with black or red ink tattoos, including photosensitivity, skin elevation and itching [5].

Nanoparticle research is currently receiving a great deal of interest due to its potential applications in biophysics, medicine, optics and electronics. A particle is generally considered to be a nanoparticle if it has dimensions below about 100 nm. For example, researchers in cancer nanotechnology are exploring methodologies to utilise functionalised quantum dots and nanocrystals to target specific tumour antigens [6]. Other medical research on nanoparticles includes the formation of a network of nanoparticles with an insulin core that can regulate and control normal blood sugar level [7]. However, despite considerable progress in nanoscience, it is often argued that the ethical and socio-legal implications of nanoparticles have been neglected [8]. Potential hazards of nanoparticles exist due to their high surface to volume ratio, which can make them very reactive [9], and their small size that can enable them to pass through cell membranes. The toxicity or biocompatibility of nanoparticles is an extremely important consideration for many of the aforementioned proposed applications. In particular carbon nanotubes, commonly used in applications such as drug delivery [10] and directed growth of neuron cells [11], have been shown to exhibit cytotoxicity potential [12], although carbon nanotube toxicity differs according to the production method used [13]. Moreover, the carbon black nanoparticles found in tattoo ink have safety profiles comparable to multi-walled carbon nanotubes [14]. Thus, there is a need to more accurately assess how tattoo ink particles directly interface with human cells and tissues. Atomic force microscopy (AFM) is one technique that can help to address this issue.

Atomic force microscopy (AFM) has been around since the mid-1980s and has become a powerful research instrument in the field of nanoscience and nanotechnology [15]. With highly specialised instrumentation and techniques, it is even possible to resolve molecular bonds [16–17]. Details of AFM operation and capabilities can be found elsewhere [18–19]. However, in brief, the AFM instrument involves a sharp probe at the end of a cantilever interacting with a surface. Not only can the AFM be used to visualise the surfaces of a wide range of materials (under various environmental conditions and over a large temperature range) the probe can also be used as a nano-indenter to ascertain mechanical properties [20] or even to carry out tensile testing of fibrils [21] or unfold protein molecules [22].

In this study we have used AFM to assess two fundamental components of skin dermis (fibroblasts and their secreted product, collagen) following interaction with tattoo ink particles. We examine the shape and size of tattoo ink particles on cellular and tissue surfaces. Further, we also investigate the cell viability of dermal fibroblasts after incubation with filtered/unfiltered diluted tattoo ink and discuss these results in the context of nanoparticle research.

## Results and Discussion

### Tattoo ink particle size distribution

**Figure 1:**
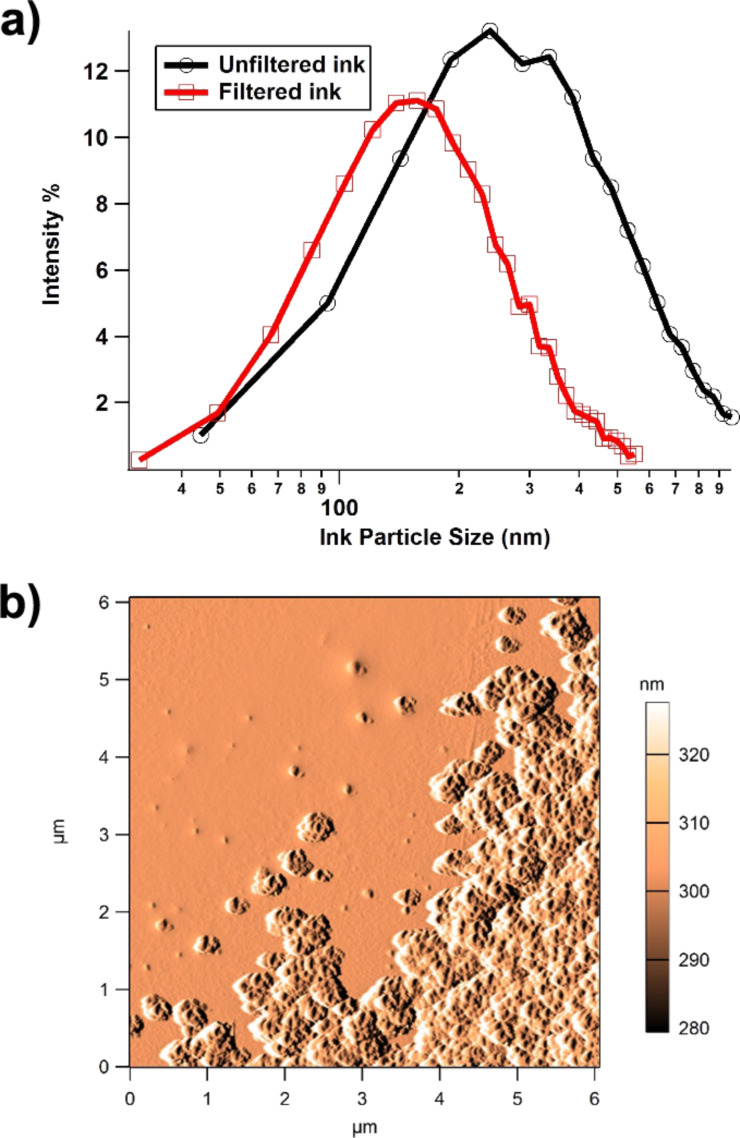
(a) Particle size distribution of filtered vs unfiltered commercially available tattoo ink, showing data ranging between 30 to 600 nm (filtered) and 40 to 970 nm (unfiltered). (b) Amplitude image of tattoo ink particles showing single and agglomerated particles adhered to a glass substrate.

Following three repeats of the particle size distribution procedure, the mode (peak intensity) value was 240.9 ± 2 nm. The same ink was also passed through a 220 nm filter to investigate the effect of increasing the proportion of nano-particulate pigment in the ink. Repeating the particle size analysis after filtration, the peak mode value reduced to 151 ± 2 nm (Figure 1a).

The light scattering technique used to make these measurements cannot distinguish between primary particles, aggregates and agglomerates. This means that the primary pigment particles may be smaller than the distribution suggests, although they cannot be larger. However, the agglomeration behaviour of the particles will be strongly influenced by the in vivo conditions in tattooing, or the in vitro conditions in cell culture. Agglomeration due to electrochemical processes can reduce the effective number of particles by orders of magnitude and this will have a profound effect on how the particles are dealt with by cells and tissues.

AFM scanning of the tattoo ink that was adhered to the glass slide was carried out in order to isolate and measure the smallest particle size, as well as to explore agglomeration behaviour. In AFM imaging, the amplitude (error) image often gives greater clarity, as it is a more efficient edge detector and is not low-pass filtered through the electronic feedback loops [23]. The *z*-scale on the amplitude images reflects changes in the height moved by the piezo sensors to maintain the engage amplitude setpoint. From the amplitude image in Figure 1b it can be seen that the particles have strongly agglomerated following the deposition process, although, a small number of individual particles can be seen in the upper left portion of the image. These single, non-agglomerated particles in the AFM image (*n* = 16) exhibited a mean projected area of 2895 nm^2^, which translates to a diameter of 60.7 nm assuming a spherical shape. For this study we have only examined one commercially available tattoo ink. However, the AFM and particle size distribution results are in strong agreement with Høgsberg et al., who carried out a large study of 58 tattoo inks of six different colours [24], where 99.94% of the volume of ink was made up from particles smaller than 100 nm.

It is clear that tattoo ink contains nanoparticles, given the peak size of the particle distribution and the AFM imaging of the ink on a glass slide. It remains unclear what potential toxicological effects tattoo ink components may have on cells, collagen fibrils etc. because of their nanometre-scale size. A gram of 60.7 nm carbon spheres would have a surface area of about 40 m^2^; over 100000 times larger than the surface area of the equivalent bulk material. In addition, materials are known to behave differently at the nanometre-level in comparison with samples at the bulk level.Nanoparticle surface atoms have an increased reactivity over bulk surface atoms [9]. However, on the whole, tattoo pigments do appear to be reasonably well tolerated by the skin, and no clear relationship between tattoo exposure and skin cancer (or cancers in general) has yet been established [24]. As cancers in general can take years if not decades of toxicant exposure to materialise, we will need to monitor how the recent dramatic increase in large-scale tattooing may impact on (skin) cancer rates.

### Microscopy of tattoo particles in skin tissue

**Figure 2:**
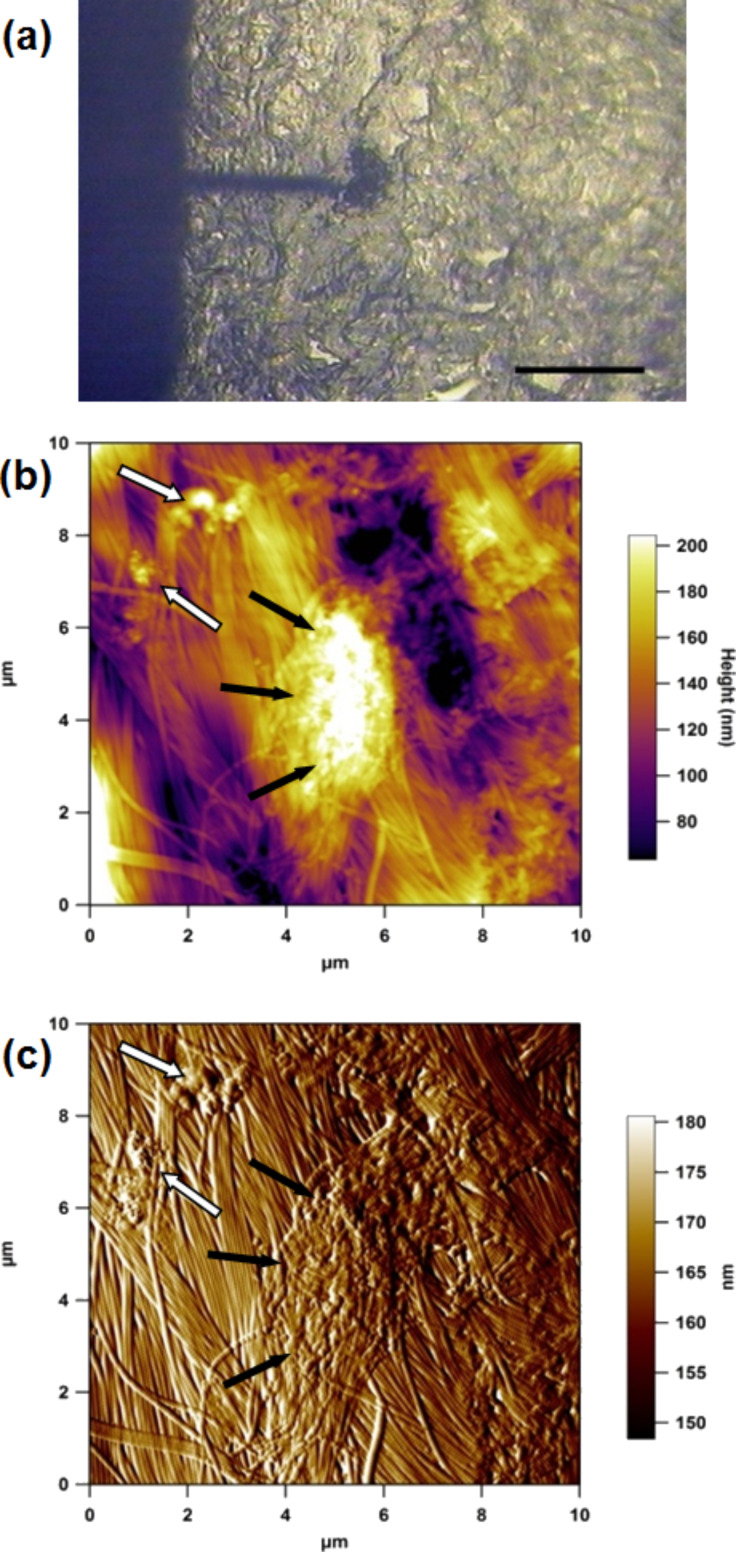
(a) AFM optical image (10×) showing the cantilever over a region of tattoo ink in the dermis; scale bar 200 μm. (b) 10 μm AFM height and (c) amplitude (error) image of cryosectioned tattooed skin. Black arrows indicate a large agglomerate and white arrows smaller agglomerates of tattoo ink particles.

Using the AFM top down optical microscope it was straightforward to manipulate the skin tissue section so that the cantilever was at the periphery of a clump of ink particles in the dermis (Figure 2a). A number of images were taken at various locations; Figure 2b shows a typical AFM height and corresponding amplitude image (Figure 2c) of a region in the upper dermis that contains tattoo ink particles. These AFM images clearly show the dense collagen fibril network with agglomerates of tattoo ink particles. The surface topography of the dermis is quite undulating with a surface roughness R_a_ of 30 nm over the 10 μm scan region.

**Figure 3:**
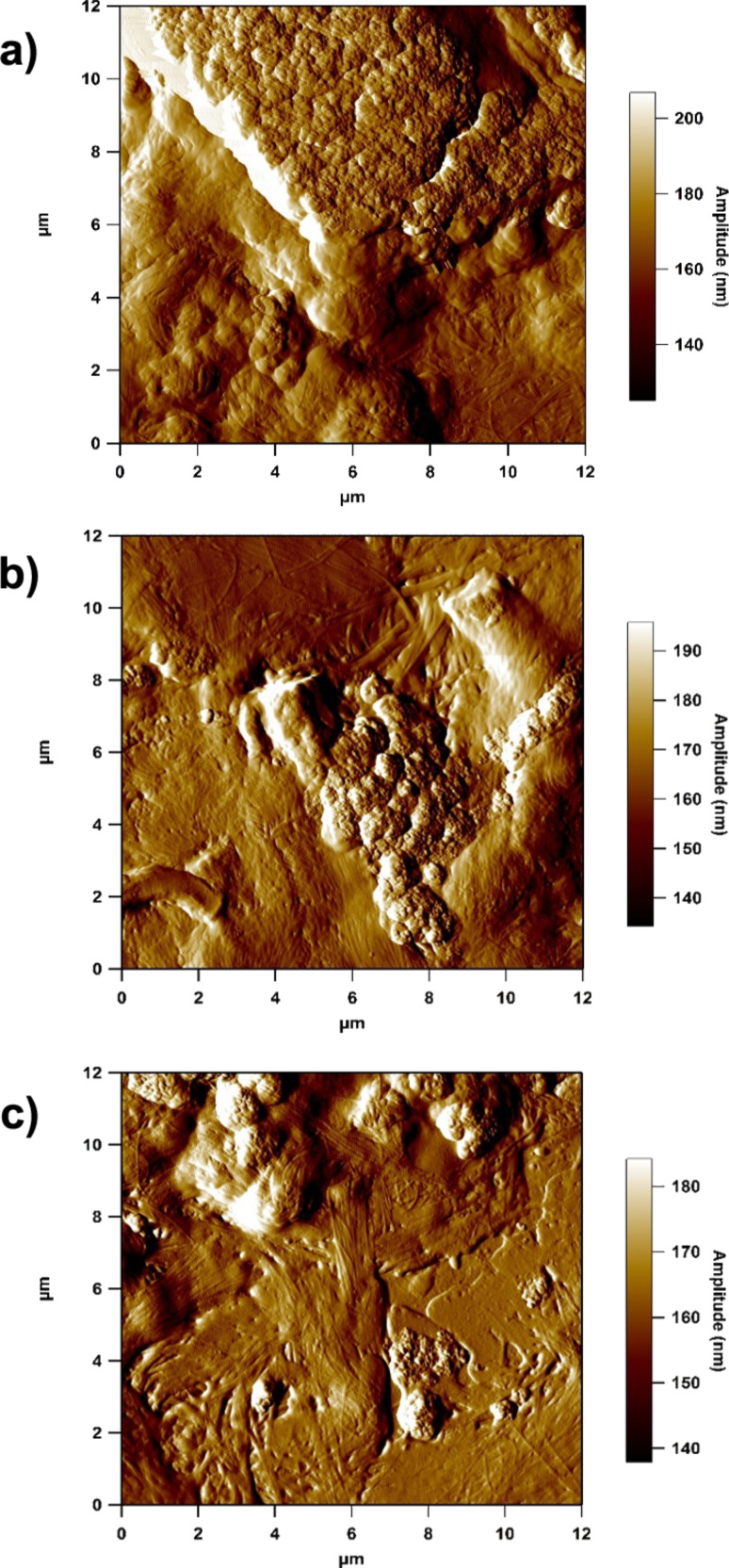
12 μm amplitude images of highly agglomerated tattoo ink particles in the collagen network.

The collagen fibrils here have a strong degree of parallel orientation, which would suggest that this region may well be scar tissue that was formed following the tattoo process. In a recent AFM study we compared scar tissue and healthy skin tissue and demonstrated that greater alignment of collagen fibrils occurs in scar tissue, as well as highlighting the reduction in the biomechanical performance of the scar tissue [25]. However, due to patient confidentiality it was not possible to find out more about how long the subject had the tattoo. Further, as the subject was 62 years old, the skin was also aged, including photo-aged from exposure of the forearm to UV irradiation. From multiple scans over a number of sections of tattooed skin tissue, it is clear that there were many regions of highly agglomerated ink particles, as shown in Figure 3. These agglomerations can be larger than the dermal cells, thereby changing the nature of the interaction between the pigment and the surrounding skin cells.

**Figure 4:**
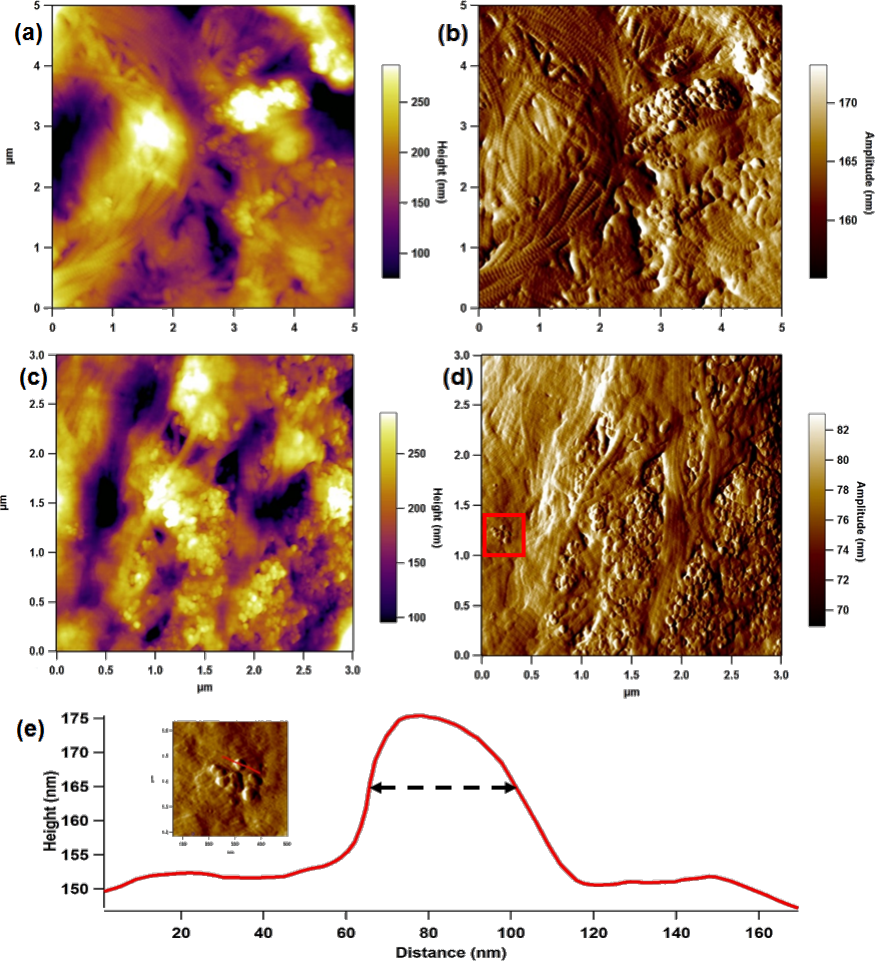
Height (a & c) and amplitude (b & d) images of disperse ink particles in the dermal collagen network. (Inset of (e)) 500 nm image of small cluster of ink particles from the solid red square (e) Line profile showing a particle of 37.5 nm width at half height.

More detailed close-up scans (Figure 4a–d) also showed ink particles in close proximity to collagen fibrils. In the amplitude images (Figure 4b and Figure 4d), the periodic banding that is associated with collagen fibrils can clearly be resolved [26–27]. The inset of Figure 4e is a detailed view of the area surrounding a small cluster of particles from Figure 4d, with the corresponding line profile shown in Figure 4e. The pigment particle here has a width of 37.5 nm at half height. When measuring a spherically shaped object with a rounded AFM probe, it is common to use the dimension at half height, to try and avoid probe convoluted distortions [28]. This line section shows that the tattoo ink pigments are truly nanoparticles embedded in the dermal collagenous network, which were visible especially at the periphery of a clump of deposited particles. Wherever primary pigment particles could be resolved they were of approximately this diameter, suggesting that the observed ink particle size distribution reflects the range of agglomeration level rather than a wide range of primary particle sizes. It is also noted that images of ink particles on the control glass slide are remarkably similar to the images of the tattoo ink in the dermis (c.f. Figure 1b and Figure 3c).

**Figure 5:**
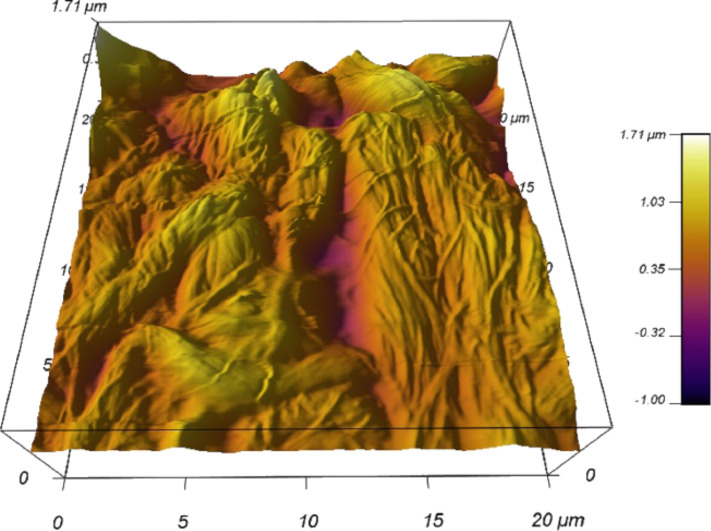
3D reconstruction of AFM image on non-tattoo portion of the skin tissue under aqueous conditions (UHQ water).

It should be noted that these images are of surfaces, sectioned from bulk samples. In vivo these particles would sit within a three dimensional extracellular matrix structure where the particles do not always sit at the surface. Further, the act of sectioning might disrupt the particles, potentially breaking up agglomerates. However, every effort was taken to avoid this by controlling the cryosectioning conditions and using new blades. Even though imaging in air may lead to unknown artefacts within the tissue, the resolution is much better. Capturing a scan of the tissue under aqueous conditions in a non-tattoo part of the skin section yielded images of collagen fibril networks without particle matter, but were of lower quality (Figure 5). AFM fluid imaging has to use a cantilever spring constant that is about two orders of magnitude lower, which makes scanning trickier. Also, the tissue surface becomes softer.

**Figure 6:**
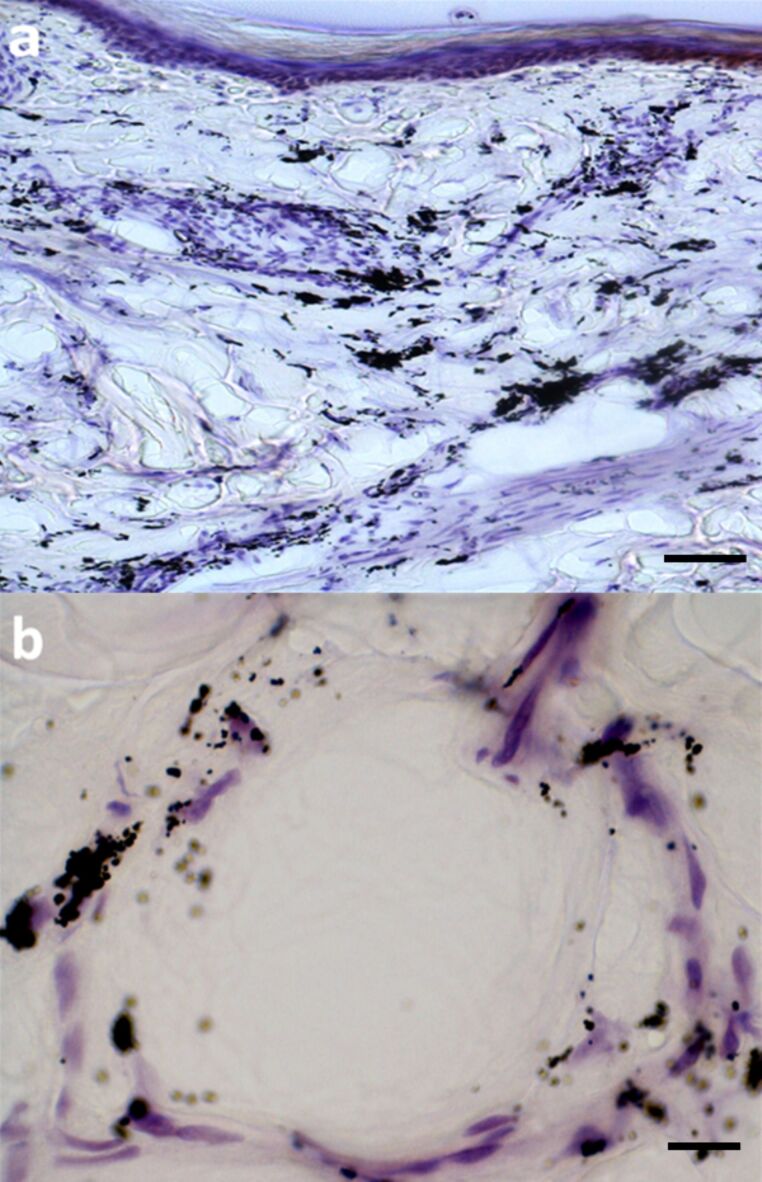
Light microscopy view of stained adult human tattooed arm skin. (a) Large deposits of dark ink particles distributed in a clumped manner in the dermis; scale bar 75 μm. (b) A deep dermal vessel with aggregations of ink particles in/around vessel wall and inside some associated cells; scale bar 15 μm.

Tattoos inevitably fade over time with a redistribution of the pigments deeper into the dermis and some even entering dermal blood vessels before transportation to local lymph nodes [29]. This leads one to question the extent of transportation of ink pigment particles throughout the body from the tattooing process. Light microscopy analysis of tattooed skin section revealed some interesting features. Figure 6a shows a transverse section of histologically stained tattooed skin, with the epidermal region uppermost in the image. Clumps of tattoo ink have dispersed throughout the upper and lower dermis. However, close inspection of a deep dermal blood vessel (Figure 6b) showed regions of tattoo ink scattered in the vessel wall as well as inside (peri)-vascular cells. A recent study, using a model system of mice tattooed with a commonly used ink to investigate the transportation and photo-decomposition of tattoo pigment particles [30], reported that the amount of ink in the mouse skin had reduced by 32 ± 16% of its initial value 42 days after tattooing. Furthermore, exposure of tattooed skin to simulated sun light and laser light also reduced the amount of ink particles retained in the skin [30]. A related study examined the distribution and accumulation of micrometre- and nanometre-sized silver particles following subcutaneous injection in rats, and found that silver nanoparticles were distributed throughout the main organs especially kidney, liver, spleen, brain and lungs [31]. By contrast, the micrometre-sized silver particles did not get into the blood circulation.

### AFM imaging of dermal fibroblasts

**Figure 7:**
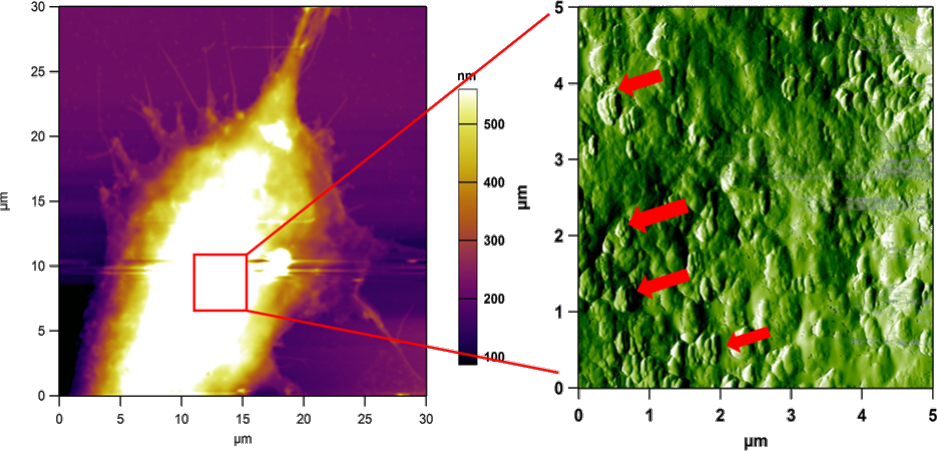
(a) AFM height image of a large fibroblast in vitro and incubated with diluted tattoo ink (1:10,000), followed by chemical fixation. (b) Phase image (5 μm) of the highest part of the cell body, which shows many regions (red arrows) of clumped ink particles on the cell surface.

Several studies have been conducted on fibroblasts using AFM to visualise both their surface and nano-mechanical properties [32–35]. Here, we show AFM images of dermal fibroblasts after incubation in diluted tattoo ink. This gives us an opportunity to visualise how tattoo ink particles may interact with dermal cells replicating the first moments following tattoo ink insertion in the skin. The AFM image of the fibroblast (Figure 7a) shows that the fixed cell is quite large, over 2 μm in height, therefore the small ink nanoparticles are difficult to see. However, phase imaging at a zoomed-in location on the cell surface (Figure 7b) highlights the ink particles very well. There appears to be a large number of ink particles attached to the cell surface in 200–500 nm clumps. However, it is not clear how much ink may have penetrated the cell, as the AFM technique can only probe surfaces.

The MTT (3-[4,5-dimethylthiazol-2-yl]-2,5-diphenyltetrazolium bromide) assay is a commonly used biological test on living cells, which broadly measures the in vitro cytotoxic effects of drugs on cell lines or primary patient cells [36]. Recently, an MTT assay for cytotoxicity assessment was carried out on fibroblasts exposed to two different diluted tattoo inks, which showed both cell death and inhibition of pro-collagen synthesis [37]. As that study was not carried out on skin fibroblasts it was decided to run a similar cell viability test using human adult skin dermal fibroblast cells (the cells targeted in skin tattooing) with both filtered and unfiltered commercially available black ink.

**Figure 8:**
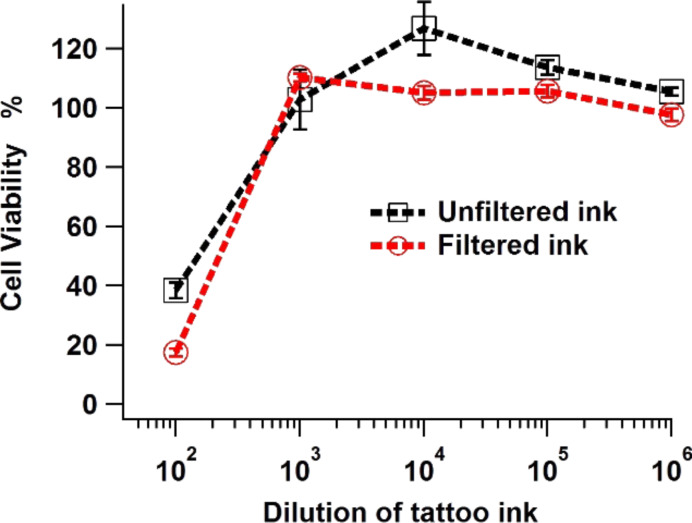
Cell viability of skin dermal fibroblasts incubated in diluted tattoo ink for seven days (filtered and unfiltered).

The MTT assay results (shown in Figure 8) indicated that at an ink dilution of 1:100 the dermal fibroblast viability was reduced significantly after a one week exposure. A reduction in viability was also noted from similar work on gingival fibroblasts exposed to a different tattoo ink source [37]. As the MTT assay is a colourimetry-based technique, the use of dark pigments can be problematic. It was found that the lowest dilution of tattoo ink for the MTT assay to work was 1 in 100. Wamer and Yin found a phototoxic effect of eight decorative tattoo inks and permanent make-up inks that contained titanium dioxide on human dermal fibroblasts [38]. The phototoxic effect from the inks was attributed to the generation of hydroxyl radicals under UV excitation.

Interestingly, fibroblast viability was lowered to a greater extent in filtered (and diluted 1:100) than unfiltered ink, i.e., 17.6 ± 1.2% viability versus 38.3 ± 2.7% viability. Filtering the ink caused larger ink particles to be removed from the suspension, reducing the overall solid content of the ink. If the cytotoxicity were simply due to the amount of pigment in the culture media then filtration would be expected to reduce toxicity. It may be that in the culture media large particles act as nuclei for agglomeration of the pigment, reducing the toxic activity. Without these nucleation sites, primary nanoparticles may remain in suspension in very large numbers, thereby presenting the cell with a greater nanoparticle challenge and so interference of their cell physiology.

This finding demonstrates that smaller pigment particles cause an increase in fibroblast death over a period of one week. A further implication of this particle size reduction may occur during attempts to remove tattoos by using lasers during which tattoo ink clusters are broken up into smaller and potentially more cytotoxic particles. It is worth highlighting that the cell incubation period of a week was used here to try and replicate the initial conditions that fibroblasts may encounter during the immediate to early post-tattoo period. The long-term behaviour of the dermal fibroblasts is not likely to be damaged or hindered and will recover following the tattoo procedure.

## Conclusion

In this study we have demonstrated that a commercially available black tattoo ink contains nanoparticles and that the modal particle size can be reduced by simple filtration. Atomic force microscopy can be used to successfully observe nanoparticles of tattoo ink in human skin tissue as well as on dermal fibroblasts in vitro. A single isolated nanoparticle of tattoo ink pigment in the region of 40 nm diameter was visualised from a dermal section of tattooed skin. The strong parallel orientation of the collagen fibrils was also noted in the tattooed skin, which was consistent with previous findings in wound healing/scar tissue [25].

Fibroblasts that were incubated in diluted tattoo ink also showed nanoparticles of ink pigments on their cell surface. Although it was not possible to explore using AFM methodology whether ink pigment nanoparticles could be taken up by the cells, this may be accomplished by ultrathin sectioning of such cells. However, the MTT assay using 1:100 diluted tattoo ink showed considerable fibroblast death. The amount of cell death with filtered tattoo ink was greater than the amount of cell death using unfiltered ink, which can be attributed to the subsequent reduction in tattoo ink particle size.

## Experimental

### Particle size distribution

The particle size distribution of the ink was determined by using a Mastersizer 3000, (Malvern Instruments Ltd., Malvern, UK). Tattoo ink (Scream Ink – Pitch Black, The Tattoo Shop, UK) was diluted in ultra-pure water (1:1000), pipetted into a disposable cuvette and then placed in the instrument. Detectors then accurately measured the intensity of light scattered by the particles in the ink for red and blue light wavelengths over a range of angles.

### Tattoo skin sample

Skin samples were obtained from the forearm of a 62 year old male. Full approval was obtained from the ethics committee of the clinic and University for the use of this tissue in this research. The skin samples were frozen upon arrival in the laboratory and stored at −80 °C. Skin specimens from the tattooed region were attached to a metal chuck using optimum cutting temperature (OCT) embedding compound (Agar Scientific, Essex, England), then sectioned in a cryostat (CM1510, Leica Microsystems, Wetzlar, Germany ) at 5 μm thickness and the sections collected onto polylysine-coated microscope slides. Tissue sections for light microscopy imaging (Nikon Eclipse 80i, Japan) were stained with haematoxylin and eosin, and then gently washed before mounting with a glass cover slip.

### Skin tissue collection for isolation and culture of dermal fibroblasts

Normal human tissue (female, 35 years of age) was sourced from elective plastic surgery (facelift) and placed immediately in transport media. After arrival at the laboratory the skin samples were cleaned in wash solution (PBS containing 5 × Pen/Strep with antimycotics/antifungal (5 × PBS)) and any fat removed. Full approval was obtained from the ethics committee of the clinic and University for the use of this tissue. Skin was cut into 0.5 × 1.0 cm pieces then placed in 0.1% trypsin overnight at 4 °C. The following day tissue was incubated for 1 h at 37 °C to separate epidermis from dermis. The epidermis was removed and the remaining dermis placed upside down in a 75 cm^3^ flask in RPMI 1640 medium (Invitrogen), then placed in a 37 °C incubator containing 5% CO_2_. After five days the dermis was removed and the culture maintained. Cells were grown in RPMI 1640 supplemented with 10% fetal bovine serum (Invitrogen), 100 μg/mL Primocin (Source Bioscience) and 1% GlutaMAX (Invitrogen).

### MTT assay

Dermal fibroblasts (passage 3) were trypsinised and seeded into 96-well plates at a density of 1 × 10^4^ cells/well. The plates were maintained overnight (16 h) in RPMI 1640 medium to allow for cell attachment. The plates were incubated for 24 h in serum-starved medium (i.e., lacking fetal bovine serum) to remove exogenous sources of growth factors, before being exposed to the tattoo ink. Unfiltered and 0.22 μm filtered tattoo ink was diluted in ‘starved’ medium and added to the plate at dilutions ranging between 1:10^2^ and 1:10^6^. The plates were placed in a 37 °C incubator containing 5% CO_2_ for 1 week, then washed with phosphate buffer solution (PBS) and incubated with serum-starved medium containing 0.5 mg/mL tetrazolium dye (MTT) for 4 h. The medium was carefully removed and 150 μL of dimethyl sulfoxide (DMSO) added to each well. The plate was gently shaken to achieve complete dissolution of the formazan crystals then the absorbance read on a spectrophotometer (Tecan Infinite) at 550 nm. The results were analysed and presented as a percentage of the untreated control samples.

A cell plate that did not undergo the MTT procedure but did have cells incubated with the tattoo ink was treated with 4% glutaraldehyde for 20 min in order to chemically fix the cells for AFM analysis. Chemically fixed samples were exhaustively washed and rinsed in ultra-pure water and gently dried under a stream of nitrogen.

### AFM

Separate microscope slides containing the cryo-sectioned skin tissue samples and fixed fibroblast cells were placed on the sample stage of the MFP-3D AFM (Asylum Research, Santa Barbara, USA) and imaged in air in intermittent contact mode using Olympus AC160 silicon probes (k ≈ 40N/m, tip radius ≈ 10 nm) and AC240 probes (k ≈ 2N/m, tip radius ≈ 10nm). The AFM optics (10×) were used to identify an appropriate region of interest before scanning.

Diluted tattoo ink (1:10,000) from the particle size testing was then deposited on to a poly-L-lysine-coated glass slide for 60 s then washed off and dried in a steady stream of nitrogen. AFM imaging was carried out to examine the tattoo ink particles that were used in the particle size distribution testing.
